# Highly Sensitive Quantitative Imaging for Monitoring Single Cancer Cell Growth Kinetics and Drug Response

**DOI:** 10.1371/journal.pone.0089000

**Published:** 2014-02-18

**Authors:** Mustafa Mir, Anna Bergamaschi, Benita S. Katzenellenbogen, Gabriel Popescu

**Affiliations:** 1 Quantitative Light Imaging Laboratory, Department of Electrical and Computer Engineering, Beckman Institute for Advanced Science and Technology, University of Illinois at Urbana-Champaign, Urbana, Illinois, United States of America; 2 Department of Molecular and Integrative Physiology, University of Illinois at Urbana-Champaign, Urbana, Illinois, United States of America; Wayne State University School of Medicine, United States of America

## Abstract

The detection and treatment of cancer has advanced significantly in the past several decades, with important improvements in our understanding of the fundamental molecular and genetic basis of the disease. Despite these advancements, drug-screening methodologies have remained essentially unchanged since the introduction of the *in vitro* human cell line screen in 1990. Although the existing methods provide information on the overall effects of compounds on cell viability, they are restricted by bulk measurements, large sample sizes, and lack capability to measure proliferation kinetics at the individual cell level. To truly understand the nature of cancer cell proliferation and to develop personalized adjuvant therapies, there is a need for new methodologies that provide quantitative information to monitor the effect of drugs on cell growth as well as morphological and phenotypic changes at the single cell level. Here we show that a quantitative phase imaging modality known as spatial light interference microscopy (SLIM) addresses these needs and provides additional advantages over existing proliferation assays. We demonstrate these capabilities through measurements on the effects of the hormone estradiol and the antiestrogen ICI182,780 (Faslodex) on the growth of MCF-7 breast cancer cells. Along with providing information on changes in the overall growth, SLIM provides additional biologically relevant information. For example, we find that exposure to estradiol results in rapidly growing cells with lower dry mass than the control population. Subsequently blocking the estrogen receptor with ICI results in slower growing cells, with lower dry masses than the control. This ability to measure changes in growth kinetics in response to environmental conditions provides new insight on growth regulation mechanisms. Our results establish the capabilities of SLIM as an advanced drug screening technology that provides information on changes in proliferation kinetics at the cellular level with greater sensitivity than any existing method.

## Introduction

Cell growth and mass homeostasis have been described as fundamental to biology, yet they are insufficiently understood [1]. This shortcoming can be traced back to the lack of methods capable of quantifying single cell mass with the required sensitivity. As a result, recently, significant progress has been made toward developing new technology for studying cell growth. These methods can be divided into *micromechanical* [2]–[4], translating resonant frequency shifts of microstructures into cell buoyancy mass, and *optical* [5]–[8], converting quantitative phase images into dry mass density maps. Optical methods are ideally suited for studying growth of adherent single cells as well as cell clusters. This capability opens up new opportunities in cancer cell biology, where sensitive measurements of cell proliferation can lead to improved understanding of the disease as well as quantitative monitoring of drug efficacy. Here we demonstrate that *quantitative phase imaging (QPI)* provides a highly sensitive method for studying cancer cell growth and for testing drugs. We use breast cancer cell lines as a model for proving the principle of our method.

Breast cancer accounts for 30% of all diagnosed cancer cases and for 14% of all cancer related deaths in women [9]. A significant decrease (7%) in incidence has been observed since 2002 that can be directly attributed to a better understanding of the link between estrogen and the growth of breast cancer[10]–[16]. This knowledge has led to the development of a class of agents that directly modulate the estrogen receptor (ER) and are now the cornerstone of treatment and prevention in ER positive patients (70% of all cases) [17]. With the realization that it is possible to exercise control over cancer growth through anti-hormones and chemotherapeutic agents came the need for large scale testing of possibly useful compounds. By 1974, over 40,000 compounds were being tested annually using murine models [18], [19]. In 1990, NCI established the primary screen of 59 human cell lines, which remains in use today [20], [21]. This new primary screen required methods to assess growth *in vitro*. Several metabolic assays were developed to measure cell growth and viability –which, to date, have remained largely unchanged [20], [22]–[26].

A widely used approach is based on the reduction of a colorless tetrazolium salt to yield a colored formozan proportional to the number of viable cells. Although such assays are useful for measuring the overall cytotoxic effectiveness of a compound, large number of cells (10^3^–10^5^) [27] have to be used to avoid incorrect conclusions from effects such as variable doubling times [20]. Furthermore, since many drugs have effects on cell cycle arrest that do not result in metabolic changes, in some cases a false readout may be made[28]. Non-tetrazolium based assays have also been developed. These assays utilize dyes that bind electrostatically to basic amino acids [25] and the measured signal is thus linear with the cell count. Despite the practical difficulties involved, one such reagent known as sulforhodamine B (SRB) was eventually adopted for routine screenings. Both assay types provide indirect measurements of growth: the tetrazolium based assays measure metabolic activity whereas the SRB assay essentially measures total protein concentration. Both methods rely on using large numbers of cells, only provide bulk measurements on the entire collection of cells, and are unable to measure time dependent responses to drugs at the cellular level. Typically, growth assay data are supplemented with fluorescence-activated cell sorting (FACS) experiments to provide additional statistical information, and study gene expression and cell cycle progression. Although very informative, FACS analyses require cells to be removed from their normal culture conditions, cell clusters to be separated, causing alterations in phenotype and morphology.

Therefore, it is becoming increasingly clear that to truly understand the nature of cancer cell proliferation and to develop personalized adjuvant therapies, there is a need for new methodologies that provide quantitative information to monitor the drug effects on cell growth and the accompanying morphological and phenotypic changes at the single cell level. The ideal drug screen should be sensitive enough to rapidly assess the response of a small number of cells to a variety of potential therapies in order to directly evaluate the response of a patient or the efficacy of a particular drug. Here we show that QPI [29] addresses this technological gap. Spatial light interference microscopy (SLIM) [30] is a QPI approach that has femtogram level sensitivity to changes in dry mass and can simultaneously provide this information at both the single cell and population level [31]. SLIM can be combined with fluorescence microscopy to measure cell cycle dependent growth [31]. In this work, we use the Estrogen Receptor (ER)-positive MCF-7 breast cancer cell line [32], [33] as a model system to demonstrate that SLIM can be used as a highly sensitive drug proliferation assay.

The MCF-7 cell line has been a widely used model for studying hormonal influence on breast cancer growth, particularly, in response to estrogens that plays a key role in promoting the growth and progression of cancer cells. We measured the growth of MCF-7 cell clusters in standard cell culture media (Veh) and under the influence of estradiol (E2), the predominant form of estrogen during reproductive years, and ICI, 182, 780 – also known as Faslodex–[34], a complete antagonist of the ER used to treat metastatic breast cancer in postmenopausal women. The results shown here establish that, in addition to being a valuable proliferation assay, quantitative phase imaging also provides biologically relevant information at the individual cell and cell cluster population level that is not accessible through other methods. Such measurements, in combination with existing molecular assays have the potential to improve drug design and characterization and to bridge the connection between our molecular understanding of cancer and drug treatment effects on cell growth and phenotypic properties such as cell size/mass.

## Results

MCF-7 cells were seeded in a two well slide chamber and measurements were performed in three cell culture conditions (see *Materials and Methods* for more details on cell culture and treatment). First, typical cell growth media as the control vehicle (Veh), second cell growth media containing 10 nM Estradiol (E2), and finally cell growth media with the antiestrogen 1 µM ICI182,780 +10 nM E2 (E2+ICI). For the E2+ICI treatment, cells were grown under E2 conditions for 10 hours before ICI was added. The 10-hour point was chosen to administer the drug since this is the earliest point at which a significant difference between the growth rates of Veh and E2-treated populations was observed (Fig. S1). A 1.55×1.16 mm^2^ area of each well was scanned every 30 minutes using a commercial phase contrast microscope equipped with a SLIM add-on module. For each experiment, one well contained an untreated control population (Veh) and the second well contained the E2 or E2 + ICI treated population. Two measurements were performed in such a manner for each condition. All data will be made freely available on request.

**Figure 1:**
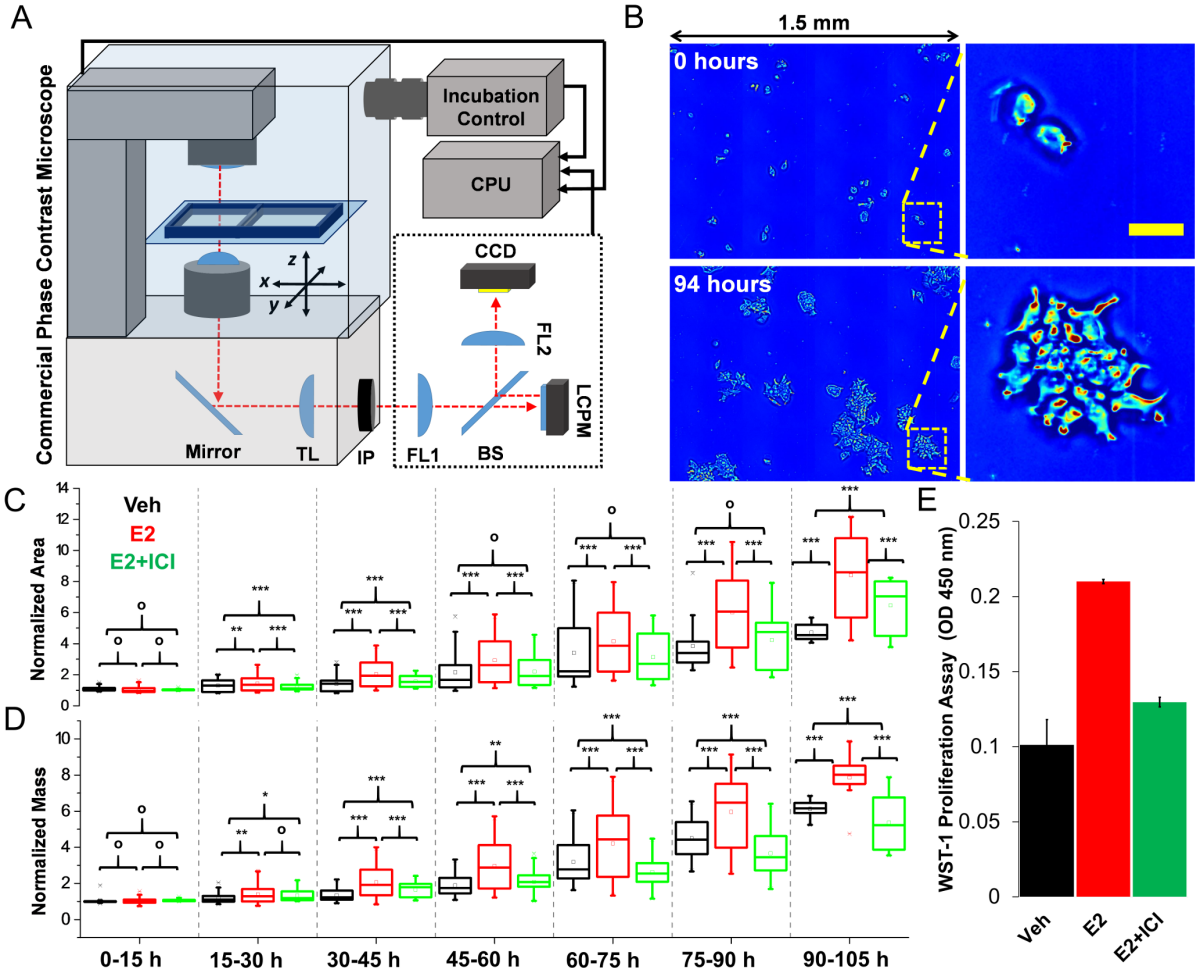
Measurement of cancer cell proliferation using SLIM dry mass measurements. (A) Schematic of experimental setup. A fully automated commercial phase contrast microscope equipped with stage top incubation control and x, y, z-scanning capabilities was used to scan a 1.5 mm×1.2 mm area in each well of a 2-well slide every 30 minutes. The components in the dotted line comprise the SLIM add-on module: Fourier Lens 1 (FL1) projects the pupil plane of the phase contrast microscope onto a Liquid Crystal Phase Modulator (LCPM), which provides control over the phase delay between the scattered and un-scattered light; Fourier Lens 2 projects the phase-modulated image onto a CCD. All components of the instrument were synchronized using the CPU. (B) Representative images of a scanned field of view in one of the chambers at 0 hours and 94 hours, the area in the dashed yellow line is enlarged and shown at each time point (yellow scale bar is 50 microns). (C) Average normalized surface area for clusters in each group in the labelled time periods. (D) Average normalized mass for clusters in each group in the labelled time periods. (C–D) Square markers indicate mean, centerline is median, top of box is 25^th^ percentile line, bottom is 75^th^ percentile line, whiskers indicate 5^th^ and 95^th^ percentiles, significance was tested using an un-paired t-test, o: p>0.05, *: p<0.05, **: p<0.01, ***: p<0.001. (E) WST-1 proliferation assay measurement at 72 hours.

A schematic of the instrument and representative SLIM images from one well are shown in Fig. 1 A and B respectively. SLIM maintains subcellular resolution (Fig. 1B) over a large area by scanning each chamber in a mosaic pattern (for more details on the measurement refer to *Materials and Methods*). From the large mosaic images, the edges of individual clusters (composed of 2–3 cells at t = 0 hours) were traced at each time point and the surface area and total dry mass were measured (see Movie S1 for a time lapse of the field of view for all groups). In this manner, we can analyze both the overall growth trends of each group and the heterogeneity at the cluster level within each population. It should be noted that this type of measurement is currently impossible to perform with any existing proliferation assay.

### Temporal Changes in Mass and Area

First, we establish the capabilities of SLIM as a proliferation assay by measuring the effects of Estradiol on the relative changes in growth, which is qualitatively similar to the information provided by conventional assays. The quantities of interest here are the relative amounts of growth in size and mass, not the absolute values. To perform this analysis, the mass and surface area of each cluster is normalized relative to its initial size, M(t)/M(t = 0) and Area(t)/Area(t = 0), respectively. The normalized area and mass for all analyzed clusters (at least 5 per group) were separated into 15-hour bins as shown in Figs. 1C and 1D. There is no significant difference in the normalized area at every time point. By contrast, the differences in the normalized mass are detectable throughout the measurement period (Fig. 1 C–D). This highlights the importance of measuring mass rather than simply the area of a cluster. Moreover, ICI treatment takes effect rapidly as the E2 group exhibits greater relative growth in the mass within 30 hours. A difference between the E2+ICI and control group can be detected by 60 hours. It should be noted that while current proliferation assays rely on using a large number of cells, SLIM measurements were performed at the individual cluster level.

### Comparison with Colorimetric Assay

**Figure 2:**
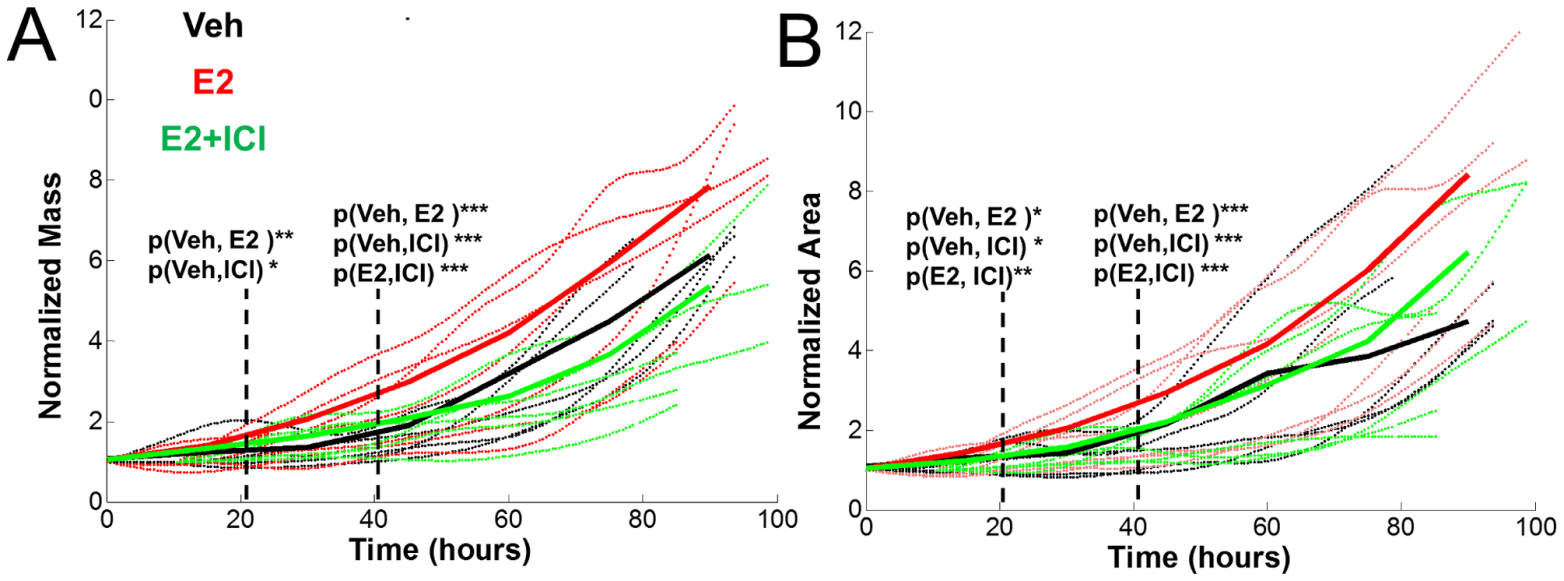
Growth data for all clusters. (A) Normalized mass vs. Time for all clusters that were analyzed. (B) Normalized area vs. time for all clusters. (A–B) Dotted lines show individual cluster data and solid lines show averaged data. Dashed lines indicate where the difference between groups becomes significant.

For comparison, measurements from a WST-1 assay taken after 72 hours of treatment are shown in Fig. 1E. It can be seen that there is a good qualitative agreement between the WST-1 data, which indirectly indicate proliferation rate, and the normalized area measurements after 75 hours. However, the normalized mass is higher in the control than the E2+ICI group after 60 hours. These differences are likely because the WST-1 assay simply measures the reduction of a tetrazolium dye outside the cell. Although the level of this reduction is related to the metabolic activity of the cells and reflects the number of viable cells in the population, it is not a direct measurement of cellular mass or size. Furthermore, such assays are restricted to providing one number for a bulk population and provide no practical way to study the heterogeneity in the population. In order to better illustrate the measured variability in the data the normalized mass and area for individual clusters are shown in Fig. 2.

### Temporal Changes in Mass Growth Rate

**Figure 3:**
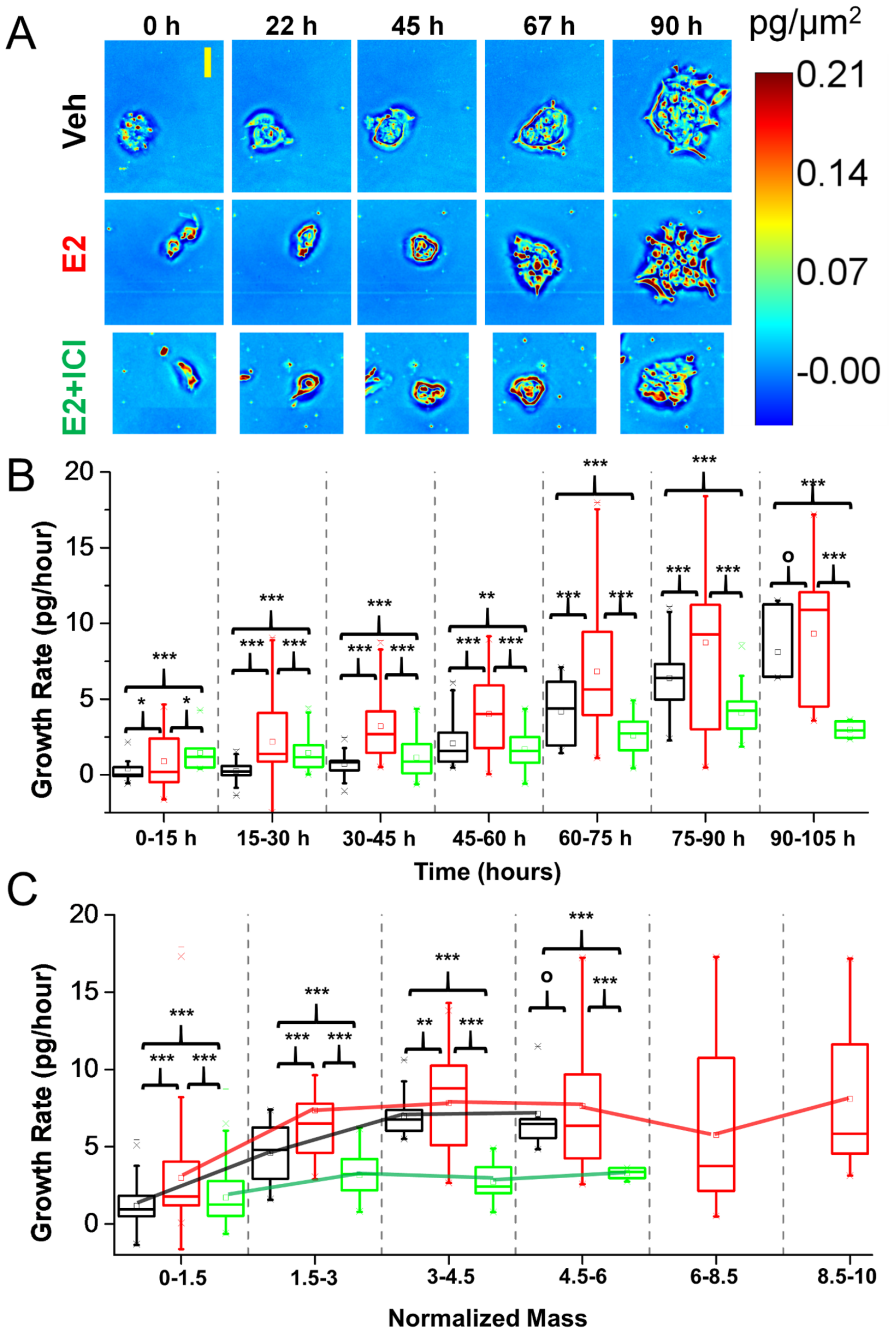
Cluster growth rate analysis. (A) Dry mass density maps of representative clusters from each group of MCF-7 breast cancer cells at every 22 hours. The colors indicate the dry mass density at each pixel as shown on the color bar. The yellow scale bar is 50 microns. Note that in the E2 + ICI group, ICI was added to each sample at 10 hours. (B) Cluster growth rate in each group in the shown time period. (C) Cluster growth rate in each group as a function of normalized mass. Solid lines are shown as a guide to the eye to determine how the growth rate is changing as a function of mass growth. (B–C) Square markers indicate mean, centerline is median, top of box is 25^th^ percentile line, bottom is 75^th^ percentile line, whiskers indicate 5^th^ and 95^th^ percentiles, significance was tested using an un-paired t-test, o: p>0.05, *: p<0.05, **: p<0.01, ***: p<0.001.

In addition to providing a quantitative understanding of how various treatments affect relative changes in size and mass, SLIM can also measure changes in the growth rate of small cell clusters as a function of time or size. Measuring the growth rate with high sensitivity is more informative than simply measuring relative changes in mass as it provides an understanding of when treatments begin to take effect and how long the effect persists. Dry mass density maps of typical clusters from each group over time are shown in Fig. 3A (see Movie S2 for). Fig. 3B shows the growth rate of clusters in each group vs. time. A significant difference in the growth rate between all three groups can be detected as early as 15 hours (5 hours after ICI treatment was administered), which is 15 hours earlier than the detectable change in both the area and mass. Furthermore, although the normalized mass is greater for the E2+ICI group than the control up to 60 hours, the growth rate of the control exceeds the E2+ICI group by 45 hours. It can also be seen that although clusters in the E2 group achieve much larger relative masses and areas than the control, there is no significant difference in the growth rates between the two groups after 90 hours.

By plotting the growth rate as a function of the normalized mass (Fig. 3C) the growth trend (exponential or linear) of the groups can be determined. It can be seen that the mean growth rate of clusters in the E2 and Veh groups continues to increase until a 4-fold increase in mass is achieved, after which the growth rate is either stable or decreases. This trend implies that for approximately two mass doublings both groups exhibit exponential growth, after which the growth is linear. In contrast, the E2+ICI group exhibits exponential growth (linear trend in Fig. 3C) until a 2-fold increase in mass is achieved, after which the growth is linear (constant curve in Fig. 3C). It is important to note here that a doubling in the mass does not necessarily correspond to a complete cell cycle as both estrogen and ICI are known to result in changes in how a cell progresses through the cell cycle, i.e., the doubling time and size and division both may be affected.

### Effects of Estradiol on Cell Size and Doubling Time

**Figure 4:**
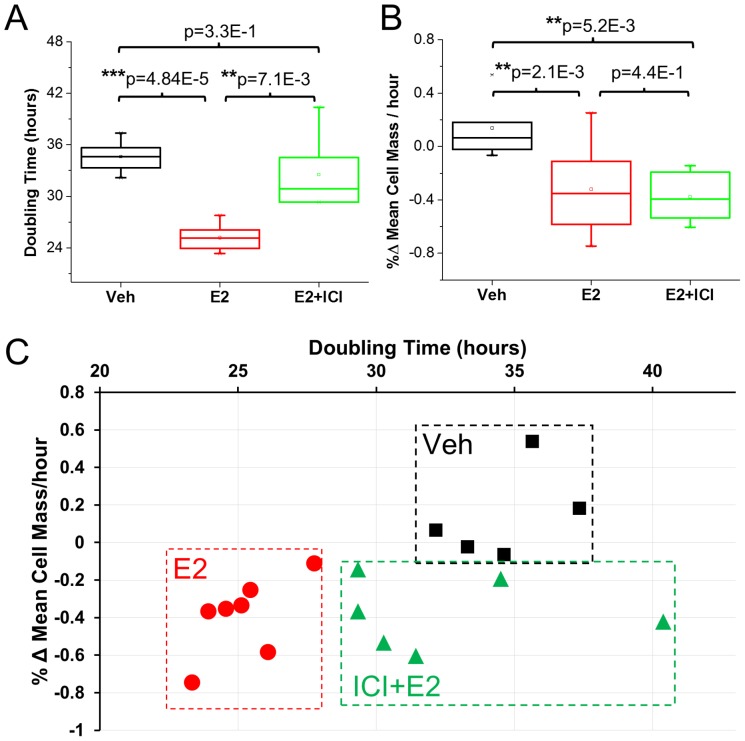
Estrogen modulated changes in proliferation kinetics. (A) Doubling time in each group, the mean doubling time is reduced by 12 hours in the E2 group compared to the Veh and E2 + ICI groups, indicating that adding ICI returns the doubling time to control levels. (B) Percent change in the mean cell mass over the measurement period for each group. A significant decrease in the cell mass is observed in both the E2 and E2+ICI groups compared to the control. (C) Measured doubling time vs. change in mean cell mass for each cluster that was measured, these two parameters can be used to separate the three groups completely and can serve as a growth signature.

To determine how changes at the single cell level contribute to the measurable changes in relative size, mass, and growth rates, we measured the doubling time and percent change in mean cell size for individual cells that compose the clusters (Fig. 4). The doubling time is calculated simply as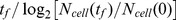, where *t_f_* is the last time point, *t_0_* is the initial time point, and *N_cell_ (t)* is the cell count at time *t*. The doubling times for the E2 group were found to be significantly lower than both the Veh and E2+ICI groups (Fig. 4A). These data show that estradiol induces cells to divide at almost twice the rate of the control group and that treatment with ICI almost completely reverses this effect. In line with previously reported studies, we find that estrogens accelerate cells through the cell cycle, resulting in an increase in cell proliferation. Of note is that the effect of ICI in increasing cell-doubling time does not imply that the cell cycle is returned to normal conditions but more likely is a result of the cells spending a larger amount of time in a specific phase of the cell cycle.

The change in mean cell mass over time was calculated by dividing the total mass of each cluster by the number of cells in the cluster. Figure 4B shows the percent change in the mean cell mass between the initial and final time points for each cluster. The results indicate a significant decrease in the mean cell mass over time in the E2 and E2+ICI cells as compared to the control. This decrease in cell mass and doubling time show that compared to the control, estrogen results in smaller, faster dividing cells, and that adding ICI results in longer doubling times along with smaller cells. The smaller cells in the E2+ICI groups imply that although the doubling time has returned to control levels, ICI is affecting the cells' metabolic activity and ability to grow normally. As shown in Fig 4C, the doubling time and change in mean cell mass provide a reliable “growth signature” for each group and suggest that the levels of expression of the estrogen receptor or presence of an ER modulator may be assessed simply by examining these two parameters. Since current assays do not provide a direct measurement of cellular growth, these are the first measurements, performed continuously on a population, which elucidate how estrogen affects MCF-7 growth kinetics at the individual cell level.

## Discussion

The results shown here establish that SLIM measurements of dry mass density can be used as highly sensitive proliferation assays. The main advantages over existing methods are that SLIM can detect changes in growth kinetics on fewer number of cells, in less time, and can be used to study differences within a population rather than just providing a number for the bulk growth of a culture. As a comparison, the WST-1 assay works with cell numbers in 10^3^–10^5^ range [27] whereas SLIM is sensitive to changes in the proliferation of even a single cell. Furthermore, SLIM provides the capability to analyze the growth rate of individual cells and clusters as a function of their mass, providing insight on the mechanism of growth regulation (e.g. whether a cell size or age checkpoint is being utilized)[35]. This information is inaccessible to any existing proliferation assay. This system can be readily used to measure growth kinetics and phenotypic effects for any cell type and treatment.

Our results also demonstrate that measuring growth at the cellular and cluster level not only provides advantages in terms of improved sensitivity and reduced sample sizes, but also yields additional information that is not accessible by existing methods. In particular, we show the kinetics and modality of action of E2 in MCF-7. Indeed MCF-7 under the influence of E2, divide faster and achieve lower masses, resulting in an increased number of cells that are on average smaller. Due to the reduced doubling time, this still results in an overall increase in total mass for E2 when compared to the control group. For cells grown in E2 and subsequently treated with ICI the doubling times returned to those found in the control, however the reduction in cell size was still greater than in the control group.

The effects of E2 and anti-estrogens on cell-cycle progression have previously been studied in detail[11], [15], [36], [37]. Both rapid and transient effects have been observed due to functional activity in both the nucleus (genomic effects) and extranuclear compartment (non-genomic effects) [38]. The rapid effects on growth are clearly observable in our data as the differences in the growth rate between the E2 and Veh exposed cells are observable after 10 hours (Fig. S1) and can be attributed to the early activation of metabolism-related genes[39]. Upon the addition of ICI, a pure ER antagonist, differences in the growth rate between the E2 and E2+ICI groups begin to appear over time (Fig. 3B). The transient effects of E2+ICI are also manifested in how the growth rate changes as a function of increase in cell size (Fig. 3C), a clear break in the growth rate can be observed when the ICI clusters approximately double in size.

Estrogens are known to activate transduction cascades that regulate many genes –both positively and negatively—that play key roles in cell proliferation and metabolism[10]–[15], [39]. It has also been shown that estrogens cause non-cycling cells (G_0_ phase) to enter the cell cycle and to rapidly progress through the G_1_ to the S phase[11], [15], [37]. This rapid progression through the cell cycle accounts for both the decreased doubling time and reduction in average cell mass observed in the E2 group (Fig. 4). On the other hand, ICI has been shown to block MCF-7 cells in the G0-G_1_ phase[15], [28], [37]. This inhibitory action on the progression through G1 is manifested in the increased doubling time of the E2+ICI group when compared to the E2 group. ICI also affects transcription of several genes responsible for growth regulation in all phases of the cell cycle. The down regulation of genes known to be responsible for proliferation in combination with the increased time spent in G1 are likely responsible for the reduction in the cell size observed in the E2+ICI group[15].

As in the case of the estrogen receptor, cell proliferation is also controlled through the activation and modulation of regulatory signaling cascades by growth factors; measuring growth at the cellular level has the potential to bridge the gap between the molecular understanding of cancer growth and actual tumor growth. Thus, it is of great interest to combine these growth measurements with fluorescence markers for cell cycle phase (as was done previously for U2OS cells [31]) or for other proteins known to play key roles in regulating proliferation. Measurement of changes in the growth kinetics as a function of the cell cycle or more specifically gene activations, will allow for better understanding of the particular action of a compound/drug on cell cycle progression.

In sum, we have demonstrated a new highly sensitive proliferation assay for drug screening applications. Although we have focused on a specific model cell system here, the experimental setup applies without alteration to other cell types and treatments. In addition to measuring growth kinetics, our method also simultaneously provides information on cellular morphology and motility [40], and can also be readily combined with other microscopy modalities. A subject of future study will be to understand and characterize the morphological differences that arise as a result of modulating the ER (see Movie S1 and S2). The biological insights provided by SLIM measurements in combination with other molecular assays will undoubtedly improve our understanding of cancer cell growth in general, and have the potential to lead to improvements in drug design, characterization, and therapeutic effectiveness.

## Materials And Methods

### Cell Culture

Commercially available MCF-7 cells were obtained from the American Type Culture Collection (Manassas, VA) and were cultured in MEM (Sigma-Aldrich Corp, St. Louis, MO) supplemented with 5% calf serum (HyClone, Logan, UT), 100 µg/ml penicillin/streptomycin (Invitrogen, Carlsbad, CA), and 25 µg/ml gentamicin (Invitrogen). Cells were then seeded in phenol-red free MEM containing 5% charcoal dextran treated calf serum to incubate for 4 days. Two chamber slides (Lab-Tek) with glass bottom coverslips were used to allow for side-by-side imaging of the control and treated samples. The medium was changed on day 2 and 4 prior to treatment with the control vehicle or ligand treatments (Estradiol (E2, 10 nM) and ICI 182,780 (ICI Fulvestrant, 1 µM)). For the colorimetric measurements, a WST1-assay (Roche, Basel, Switzerland) was used and absorbance was measured at 450 nm using a BioRad 680 Microplate Reader.

During imaging the cells were kept at 37°C and in a 5% CO2 atmosphere with an incubator and heated stage insert. Each well was scanned every 30 minutes in a 4×4-tile pattern with a Zeiss EC Plan-Neofluar 10×/0.3 PH 1 objective providing a total field of view of 1.55×1.16 mm^2^. A z-stack of 12 slices (48 µm) was recorded at each position. The exposure time was 15 ms for each image at full lamp power (3,200 K, or 10.7 V). For each experiment, one well was left untreated to serve as a control population and the other well was treated with either the E2 or E2+ICI condition. Each condition was measured along with its control two times.

### Imaging

Imaging was performed using spatial light interference microscopy (SLIM)[30], [41]. SLIM is a white-light optical interferometry modality designed as an add-on module to a commercial phase contrast microscope. In brief, SLIM operates by projecting the back focal of a phase contrast objective onto a liquid crystal phase modulator (LCPM). The LCPM is calibrated to precisely shift the phase of the light scattered by the sample relative to the un-scattered light. Intensity maps are recorded at phase shifts of zero, π/2, π, and 3π/2.

The SLIM setup used in this study is based on a Zeiss Axio Observer Z1 (Zeiss catalog # 431007901000) motorized inverted research imaging microscope. The microscope base is equipped with a motorized focus drive (minimum step width 10 nm). The objectives used for this study is a Zeiss EC Plan-Neofluar 10×/0.3 PH 1 M27. The intermediate image following the objective and tube lens is directed to left port of the microscope where the SLIM module is attached. The intermediate image is magnified by a 4f system with a focal length 150 mm doublet (Thorlabs, AC508-150-A1-ML) and a focal length 200 mm doublet (Thorlabs, AC508-150-A1-ML). A second 4f system following the magnifying system is comprised of a Fourier lens L1 (doublet with focal length 300 mm, Thorlabs, AC508-300-A1-ML) and Fourier lens L2 (doublet with focal length 500 mm, Thorlabs, AC508-500-A1-ML). The LCPM (array size 7.68 mm×7.68 mm, Boulder Nonlinear, XY Phase series, Model P512-0635) is placed at the back focal plane of L1 and is thus overlaid with the back focal plan of the objective. A polarizer (Edmund Optics, Stock # NT47-316) is placed in front of the LCPM to operate it in phase modulation mode. A Zeiss AxioCam MRm (1388×1040 pixels, pixel size 6.45 µm×6.45 µm, Zeiss catalog #4265099901000) is used for image acquisition. The SLIM module adds 2.22× magnification to the intermediate image. The microscope is also equipped with live cell environmental controls optimized for long time studies, including incubator XL S1 W/CO2 kit (Zeiss catalog #1441993KIT010) and a heating insert P S1/Scan stage (Zeiss catalog #4118609020000). The microscope is automatically controlled by AxioVision (Zeiss catalog #4101300300000) with multi-position, time-lapse, mosaic and Z-stack acquisition capabilities. The LCPM is automatically controlled using Labview. Matlab and ImageJ are used for image processing and visualization. Four intensity maps are recorded in this manner at phase shifts of zero, π/2, π, and 3π/2. Remarkably, since the initial calibration of the system the accuracy has remained stable and thus no re-calibration or adjustment is required between experiments.

### Data Analysis

The quantitative phase image is reconstructed from the four phase shifted intensity images as:
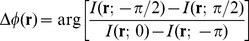
The dry mass density at each pixel is calculated as 
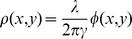
 where γ = 0.2 ml/g is the refractive increment of protein, λ is the center wavelength of the illumination and φ is the measured phase difference [31]. The dry mass density map is calculated at each z-location, and the projected maximum of the density is then calculated over the entire stack. The total mass is then calculated by integrating the dry mass density map over a region of interest. It should be noted that the accuracy of the dry mass measurement might be affected by debris present in the culture. Although this was not necessary for any of the experiments in this work, this affect may be mitigated by periodically replacing the culture media. The noise in the dry mass measurements due to such affects is shown in Fig. S2.

For the cluster analysis in this study the images were manually segmented using ImageJ and cells were counted at the beginning and end frames of each time series. In cases where the culture reaches confluence segmentation may become prohibitively difficult, thus it is important to control the initial seeding density to avoid this. The dry mass data was then smoothed using a cubic smoothing spline. For the binned data shown in Fig. 1 and 2, the smoothed data was sorted according to the variable over which the binning is performed. At least five clusters were analyzed per group.

## Supporting Information

Figure S1 **E2 vs. Veh.** (A) Average dry mass (left axis, solid lines) and WST-1 assay data (right axis, dashed lines). (B) Growth rate vs. Time, a significant shift between E2 and Veh can be seen at 10 hours.
(TIF)

Figure S2 **Sensitivity of dry mass measurement.** Due to debris in the field of view, the noise in the mass measurement is higher (∼pgs) than SLIM's capabilities (∼fgs).
(TIF)

Movie S1 **SLIM images from Veh, E2 and E2+ICI experiments.** A clear difference in the growth rates and size of clusters is apparent.
(WMV)

Movie S2 **Dry mass density maps across time of representative clusters from Veh, E2 and E2+ICI experiments.**
(WMV)
