# Supplementary figures and images for: Highly Sensitive Quantitative Imaging for Monitoring Single Cancer Cell Growth Kinetics and Drug Response
Source: PLoS One. 2014 Feb 18;9(2):e89000. doi: 10.1371/journal.pone.0089000 (PMC3928317; doi:10.1371/journal.pone.0089000)

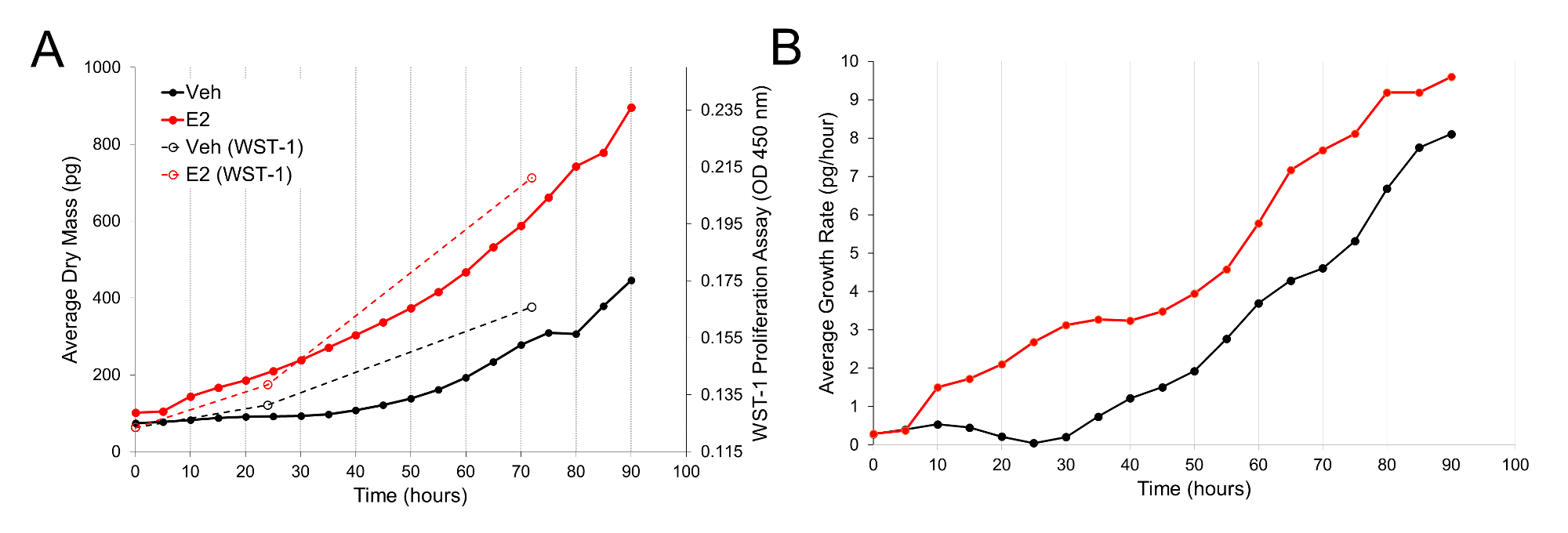

Supplement: Figure S1 — E2 vs. Veh. (A) Average dry mass (left axis, solid lines) and WST-1 assay data (right axis, dashed lines). (B) Growth rate vs. Time, a significant shift between E2 and Veh can be seen at 10 hours. (TIF) [file pone.0089000.s001.tif]
